# Kin selection explains the evolution of cooperation in the gut microbiota

**DOI:** 10.1073/pnas.2016046118

**Published:** 2021-02-01

**Authors:** Camille Simonet, Luke McNally

**Affiliations:** ^a^Institute of Evolutionary Biology, School of Biological Sciences, University of Edinburgh, Edinburgh EH9 3FL, United Kingdom;; ^b^Centre for Synthetic and Systems Biology, School of Biological Sciences, University of Edinburgh, Edinburgh EH9 3BF, United Kingdom

**Keywords:** cooperation, comparative analysis, microbiome, evolutionary microbiology

## Abstract

Through the secretion of “public goods” molecules, microbes cooperatively exploit their habitat. This is known as a major driver of the functioning of microbial communities, including in human disease. Understanding why microbial species cooperate is therefore crucial to achieve successful microbial community management, such as microbiome manipulation. A leading explanation is that of Hamilton’s inclusive-fitness framework. A cooperator can indirectly transmit its genes by helping the reproduction of an individual carrying similar genes. Therefore, all else being equal, as relatedness among individuals increases, so should cooperation. However, the predictive power of relatedness, particularly in microbes, is surrounded by controversy. Using phylogenetic comparative analyses across the full diversity of the human gut microbiota and six forms of cooperation, we find that relatedness is predictive of the cooperative gene content evolution in gut-microbe genomes. Hence, relatedness is predictive of cooperation over broad microbial taxonomic levels that encompass variation in other life-history and ecology details. This supports the generality of Hamilton’s central insights and the relevance of relatedness as a key parameter of interest to advance microbial predictive and engineering science.

## Significance

This is a comparative study attempting to explain the pattern of cooperation across a number of microbial species. Hamilton’s inclusive-fitness theory makes the very general prediction that increased genetic relatedness should drive the evolution of cooperation. Various arguments have dismissed the validity of this prediction in microbes, but without ever testing the broad taxonomic support for those arguments. Here, we rehabilitate the central role of relatedness by showing that its power to predict cooperative gene content holds across the full diversity of the human gut microbiota. Explaining broad-scale patterns is critical to a unifying variable for predictive science and broad applications. The manipulation of relatedness may offer an opportunity to engineering microbial communities, such as the gut microbiota.

Managing complex microbial communities (MCs) is key to a range of applications in the midst of our society’s challenges from microbiome manipulation (1) to sustainable food production (2) and climate regulation (3). The successful engineering of such communities requires the field of MCs and microbiome research to advance into more predictive science (4, 5). Crucial to this are theories of broad predictive ability. Firstly, such theories allow predictions that consistently hold across the vast diversity of microbial species making up those communities, and, secondly, they facilitate the translation of theory into actionable tools.

Cooperative interactions are central to microbes’ lives, as well as how they interact with and modify their environment (6–13). Through the secretion of “public goods,” such as toxins, enzymes, or signaling molecules, microbes cooperatively exploit and modify their habitat (14, 15). Recent “omics” studies have demonstrated the important role of such cooperative interactions in the evolution and function of real communities (16, 17), including diseases-associated communities (18). To predict and engineer the dynamics and evolution of MCs, it is therefore essential to understand the factors having a broad influence on the evolution of cooperation in the species making up these communities.

**Fig. 1.**
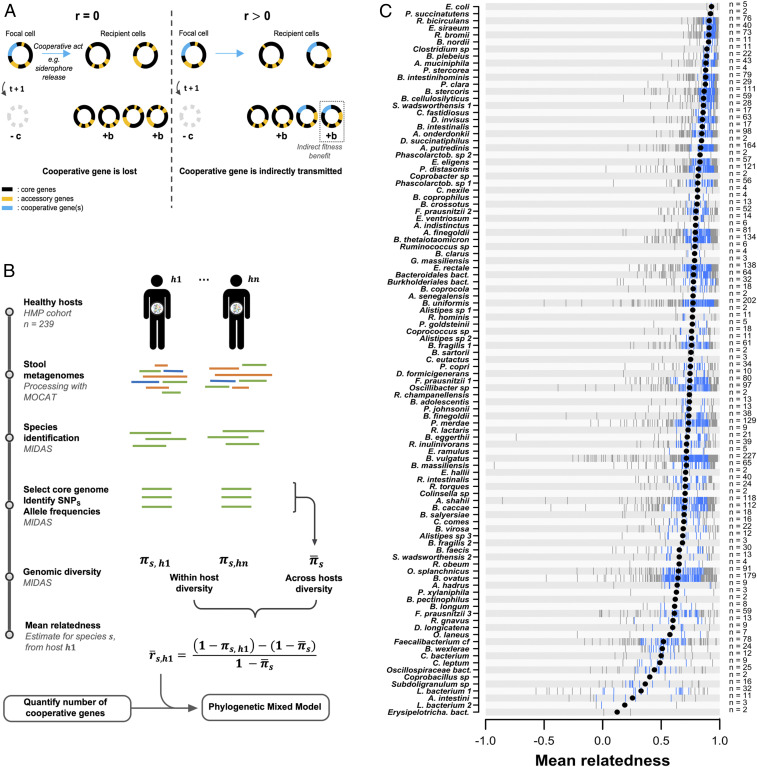
Genetic relatedness in the human gut microbiome. (*A*) Schematic illustration of indirect fitness benefits. The cooperative cell loses the opportunity to produce *c* daughter cells (cost *c*). The help provided to the recipient cells allows them to each produce an additional *b* daughter cells (benefit *b*). The cooperative genes of the altruist cell are “indirectly transmitted” if the benefits provided enhance, on average, the reproduction of cells that also carry those cooperative genes, i.e., are genetically related; *r* > 0. (*B*) Methods schematic summary. Detailed within- and across-samples core genome size and nucleotide diversity are given in Dataset S1. SNPs, single-nucleotide polymorphisms. (*C*) Relatedness measures obtained for 101 species of the human gut microbiome. Vertical ticks are single point estimates of relatedness. The number of point estimates (i.e., number of hosts within which each species was found) is indicated on the right. The black dots represent the mean. Blue ticks are values between 25% and 75% quantiles.

How cooperation evolves is puzzling because populations exhibiting such behavior are at risk from invasion by selfish cheats, reaping the reward without paying any of the cost (19). Hamilton’s kin-selection theory provides an explanation: Even if sacrificing its own reproduction by helping a close relative reproduce, a cooperative individual can still pass on its genes to the next generation, albeit indirectly (20). Therefore, altruism is favored when fitness costs to the helper are overcome by benefits provided to the recipient weighted by their genetic relatedness (*rb* > *c*, “Hamilton’s rule”). This gives a central role to genetic relatedness, because it limits those indirect fitness benefits (21) (Fig. 1*A*). Hamilton’s theory generates a prediction of great generality: All else being equal, increased relatedness should lead to more cooperation. Contrary to predictions based on specific mechanisms [e.g., pleiotropy (22) or greenbeard genes (23, 24)] or that apply to a limited amount of taxa [e.g., particular scenarios calling upon preadaptations (25, 26)], the generality of Hamilton’s prediction is useful in that it identifies a unifying parameter (27). In the context of mastering MCs that are hugely diverse, such unifying principle is key. The question is then whether this is true in practice: Is relatedness broadly predictive of the evolution of cooperation in microbes?

Although kin selection has been a leading explanation for the evolution of cooperation from microorganisms to vertebrates in the field and in the laboratory (12, 13, 19, 23, 24, 28–33), three main arguments cast doubt on its generality and predictive power in microbes. Firstly, even if relatedness drives cooperation, the direction of its effect may depend on the details of the biology of a particular cooperative behavior. For example, it has been shown that when a public good can be partly privatized (e.g., with strain-specific receptors), the public good becomes a competitive trait, therefore leading to a negative relationship between relatedness and the level of public-good production (34). Such variability in the direction of effect means that prediction may not be consistent across different types of cooperative behavior and species. Secondly, it has been suggested that interspecies interactions (i.e., when public goods provide interspecific benefit) may render relatedness unimportant at driving cooperation within species. This has been observed in the production of siderophores (a secreted iron-scavenging molecule acting as a public good) in *Pseudomonas aeruginosa*. In conditions such that siderophores also provided cross-species benefits (environment detoxification), the addition of a compost community allowed the growth of noncooperators, irrespective of the level of relatedness (35). This challenges the effective importance of relatedness in real-world, complex communities. Third, theoretical work predicts that the population-genetics effects at work in the kin-selection framework may be unimportant in microbes owing to strong selection (25, 36, 37). Together, these arguments suggest that intraspecific relatedness may have minor or idiosyncratic effects on the evolution of cooperation in microbes.

Although these studies highlight potential limitations in the power of relatedness to predict the evolution of microbial cooperation, they do not assess their actual importance across the microbial tree of life. The ultimate test of the broad role of relatedness in the evolution of cooperation is to use a comparative analysis to assess whether relatedness can predict the phylogenetic distribution of cooperative traits. While such studies exist for a range of animal species [shrimps (38), mammals (39, 40), birds (41), and Hymenoptera (42, 43)], none have been performed in microbes. Conducting a comparative analysis in microbes is more than a mere additional test of Hamilton’s rule. Microbes constitute an excellent system to test the claim of generality: It assesses relatedness predictive power over a broad set of ecological idiosyncrasies by 1) including a large number of phylogenetically distant species with different ecology, 2) comparing a variety of cooperative behaviors that have very different ecological contexts (while most existing studies focus on a single cooperative behavior), and 3) using actual genomic relatedness from sequencing data, rather than of proxy such as promiscuity level (41).

We conducted such phylogenetic comparative analysis across the full diversity of the human gut microbiota, encompassing 37 genera, testing the effect of relatedness on six different forms of microbial cooperation.

## Results

### Relatedness and Cooperation in the Human Gut Microbiota.

Using 239 healthy human stool metagenomes (44), we computed relatedness for a large diversity of species in the gut microbiome. We identified 101 species (37 genera) meeting minimum coverage and prevalence requirements for this analysis. In this selected set of species, the mean relative abundance ranged from 0.004 (*Collinsella_sp_62205*) to 0.26 (*Prevotella_copri_61740*) and was detected in 2 to 227 hosts (see Dataset S1 for per-species-per-host details). Relatedness is a statistical measure of the genetic similarity between interacting individuals (potential beneficiaries of the altruistic behavior) relative to the average population-wide genetic similarity (competing individuals). Through their effect on their host (5, 45) (e.g., host immune-system modulation), bacteria potentially interact with each other at the scale of the whole host. In parallel, bacterial strains spread over large geographic areas (46, 47) and colonize hosts in various and dynamic assemblages (48), meaning that competition can occur globally across hosts. Therefore, we capitalized on strain-level analysis tools (49) to compute the genomic similarity within a host and across all hosts to calculate the genetic relatedness (Fig. 1*B*). We obtained estimates of relatedness for all observed host–species pairs and found that the vast majority of the gut-microbiome species (>90% of the species included in this analysis) had an average relatedness greater than 0.5 (Fig. 1*C*). This means that for most gut-microbiota species, the conspecific they potentially interact with within their host is at least as related as siblings in sexually reproducing species.

We then assessed each microbial species’ propensity for cooperation, for six broad classes of bacterial cooperation. First, secreted products (henceforth referred to as “secretomes”) can be seen as cooperative from the producing cell’s perspective, either because their kin can benefit directly from it (public good) or because of the reduced competition that they create if they have antagonistic effects on other microbes (50). Second, biofilm, quorum-sensing, siderophores, and antibiotic-degrading enzymes are four well-described forms of bacterial cooperation. Finally, secretion systems can be perceived as a sixth cooperation class, as it differs from the secretome in that it captures genes coding for structural cellular components involved in secretion, rather than secretions themselves.

**Fig. 2.**
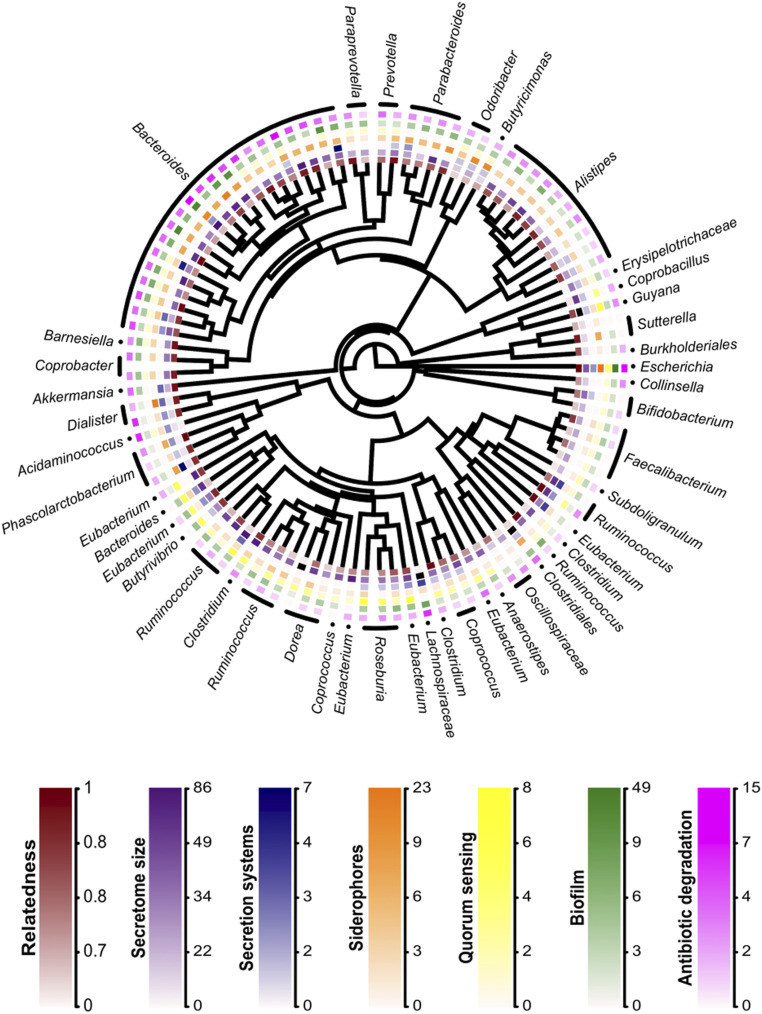
Genetic relatedness and cooperation across the gut-microbiome phylogeny. Relatedness is the mean genetic relatedness. The secretome is the number of protein-coding sequences coding for a secreted product. The five other forms of cooperation are measured as the number of protein-coding sequences annotated with a GO term falling in that cooperation category. *n* = 101.

The kin-selection framework can be applied to understand the build-up of a genome, with species having higher relatedness expected to carry more social genes (Fig. 1*A*; *SI Appendix*, *SI Text*). Therefore, we measured microbe cooperativity on the basis of their genome content, by quantifying their number of genes falling in those six classes of cooperation (Fig. 2). For the secretome, we used a sequence-motif-based localization-prediction tool to count the number of protein-coding sequences coding for secreted products. For the five other measures, we used gene ontology (GO) annotations (Dataset S1 and *SI Appendix*, Figs. S1–S5).

In each case, the number of genes for a given class of cooperation is a measure of the number of cooperative phenotypes in that class. For example, for the secretome, two secretome genes mean that there are two different secreted proteins, which are likely to be different public goods. For other classes of cooperation (GO measures), more than one gene may be necessary for expression of the phenotype (e.g., pyoverdine biosynthesis involves 14 genes), but more siderophore genes are still likely to mean production of more than one siderophore (e.g., producing pyoverdine and pyochelin, which would be two distinct cooperative phenotypes).

### Hamilton’s Theory Predicts Cooperative Gene Content Evolution in Gut-Microbe Genomes.

We tested for an association between relatedness and cooperation, for each form of cooperation, using Bayesian phylogenetic mixed models (Poisson response model, *n* = 101 species, 37 genera). Our modeling accounts for potential nonlinear scaling of the number of cooperative genes with genome size, which simply arises from a gene-sampling process in a genome with a set of constant essential genes (*SI Appendix*, *SI Text*).

**Fig. 3.**
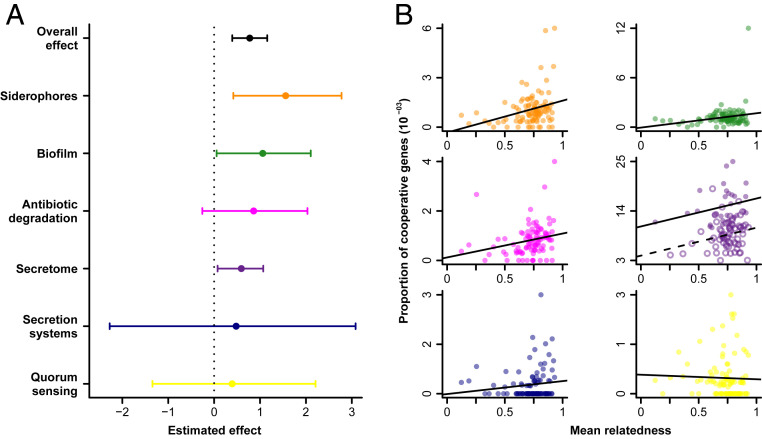
Genetic relatedness predicts cooperation in gut microbes over several forms of cooperation. (*A*) Total regression coefficient estimates of cooperation on relatedness. The dot and horizontal bar represent the mean and the 95% credible interval of the posterior distribution for each phylogenetic mixed model. The “overall effect” is the estimate obtained from the random-effects meta-analysis (mean and 95% CI). (*B*) Proportion of cooperative genes as a function of mean relatedness for each form of cooperation. Lines are ordinary least-square trend lines to illustrate the trends. The secretome panel shows separate trend lines for Gram-positive (open circles and dashed lines) and -negative (filled circles and solid lines).

For secretome size, we found a significant positive effect of relatedness (β=0.59, 95% credible interval [$CI_{95}$] =0.08,1.07; $P_{MCMC}=1.9\times 10^{-2}$; Fig. 3; *SI Appendix*, Table S1). Biologically, this coefficient means that after controlling for genome size, we predict a 60% increase in the number of genes coding for secreted products between the gut-microbiome species with the lowest measured relatedness (*Erysipelotrichaceae bacterium*; *r* = 0.12) and the species with the highest measured relatedness (*Escherichia coli*; *r* = 0.93). We also found a significant positive effect of relatedness on the number of genes coding for cooperation for siderophores and biofilm classes (respectively: siderophores: β=1.56, $CI_{95}=0.42,2.78$; $P_{MCMC}=9.5\times 10^{-3}$, biofilm: β=1.06, $CI_{95}=0.06,2.10$; $P_{MCMC}=4.6\times 10^{-2}$; *SI Appendix*, Table S1). There was no significant effect for quorum-sensing, secretion systems, and antibiotic degradation (respectively: quorum-sensing: β=0.39, $CI_{95}=-1.34,2.21$; $P_{MCMC}=6.7\times 10^{-1}$; secretion systems: β=0.48, $CI_{95}=-2.27,3.08$; $P_{MCMC}=7.2\times 10^{-1}$; antibiotic degradation: β=0.86, $CI_{95}=-0.26,2.03$; $P_{MCMC}=1.4\times 10^{-1}$; *SI Appendix*, Table S1).

Our purpose here is to generalize conclusions about the importance of relatedness, both across the microbial diversity, but also across various forms of cooperation that have different ecological and evolutionary constraints. Here, our measure across six distinct forms of cooperation successfully captured distinct sets of genes (Dataset S1 and *SI Appendix*, Fig. S6). Therefore, we used a random-effects meta-analysis across the different forms of cooperation to obtain a global estimate of the effect of relatedness on the cooperative gene content, with a CI accounting for the certainty in each class-specific estimate. With this approach, we found a significant global effect of relatedness on the number of genes involved in cooperation (β=0.78, Std.error=0.194, z-value = 3.99, $CI_{95}=0.40,1.16$; $P_{MCMC}=6.5\times 10^{-5}$; *SI Appendix*, Table S6). These results hold when accounting for the uncertainty in relatedness estimates (*SI Appendix*, *SI Text* and Tables S2 and S5).

### Organismal Ecology and Relatedness in the Gut Microbiome.

**Fig. 4.**
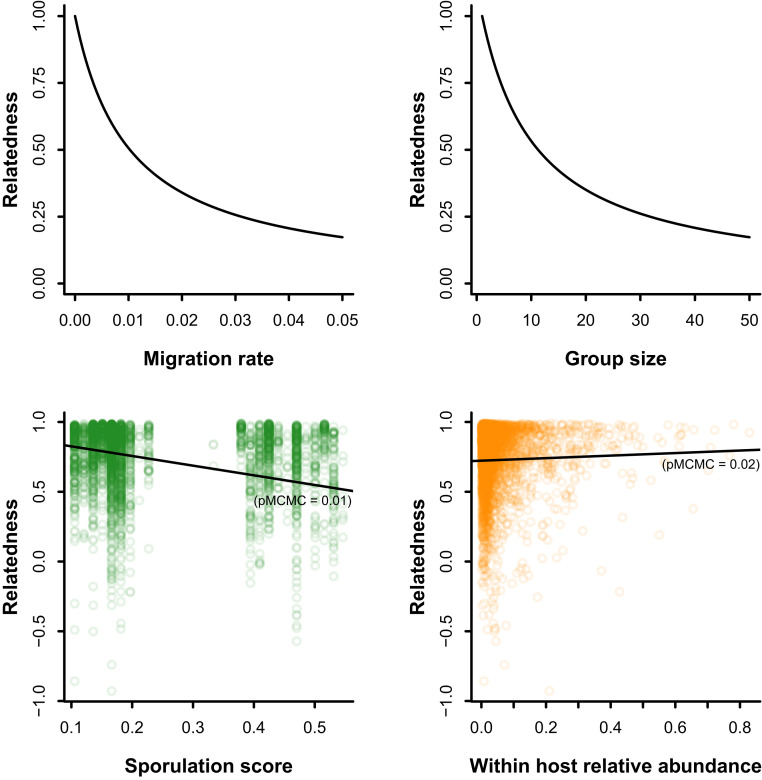
The ecological drivers of relatedness. (*Upper*) Theoretical predictions for the effect of migration rate and group size on relatedness in the infinite island model. Theoretical equilibria were derived by from El Mouden et al. (2010) (52). (*Lower*) Observed effects of sporulation (proxy for migration) and relative abundance (proxy for group size) on the genetic relatedness in the human gut microbiome. Regression lines and pMCMC shown are from the phylogenetic mixed model of within-host relatedness with sporulation score and within-host relative abundance as fixed predictors (*n* = 3,874).

Relatedness itself is likely shaped by the ecological dynamic of species. Classically, the infinite-island model predicts that in an infinite or very large number of demes all connected by migration, relatedness should decrease with both group size and migration (51, 52) (Fig. 4, *Upper*). We constructed a Bayesian phylogenetic mixed model of within-host relatedness to test these predictions over the pattern of relatedness we measured across the human gut microbiota.

Adaptations facilitating migration should correlate with gut-species migration rate. The ability to form spore is an adaptation that allows efficient dispersal of the organisms through the environment and among hosts (53). Hence, we computed sporulation scores as a first-order proxy for migration rate. We used within-host relative abundance to account for group size. In agreement with the predictions of the infinite-island model, we found a negative relationship between sporulation scores and relatedness (β=0.69, $-CI_{95}=-1.25,0.10$; $P_{MCMC}=1.5\times 10^{-2}$; Fig. 4, *Lower Left*; *SI Appendix*, Table S3). However, we found a positive relationship between relative abundance and relatedness (β=0.09, $CI_{95}=0.008,0.17$; $P_{MCMC}=2.5\times 10^{-2}$; Fig. 4, *Lower Right*).

### Relatedness Holds the Same Effect on Cooperation after Accounting for the Ecological Factors Shaping It.

Given their significant effects on relatedness, it is possible that group size and migration rate could be the drivers of the apparent effect of relatedness on cooperation via some alternative mechanism. We tested this by including sporulation score and mean relative abundance in our phylogenetic mixed models as predictors of cooperation. We found that relatedness remained significantly predictive (with similar effect size) of microbe cooperative gene content after controlling for sporulation scores and relative abundance (results for the models for each form of cooperation reported in *SI Appendix*, Table S4; random-effect meta-analysis over the six models: $\beta_{mean\;relatedness}=0.74$, Std.error=0.20, z−value=3.75, $CI_{95}=0.35,1.13$; $P_{MCMC}=1.78\times 10^{-4}$; *SI Appendix*, Table S6).

### A Mix of Direct and Indirect Effects.

Hence, we find that these two ecological factors shape relatedness, but that relatedness retains the same predictive power after controlling for these ecological factors. The model also shows that both relative abundance and sporulation score have themselves a positive significant effect on quorum-sensing and a marginal effect on antibiotic degradation (*SI Appendix*, Table S4). The meta-analysis model suggests an overall marginal effect of relative abundance on cooperation, but no effect of sporulation ($\beta_{relative\;abundance}=1.79$, Std.error=1.02, z−value=1.75, $CI_{95}=-0.21,3.78$; $P_{MCMC}=8.00\times 10^{-2}$ and $\beta_{sporulation\;score}=-0.30$, Std.error=0.69, z−value=−0.44, $CI_{95}=-1.66,1.05$; $P_{MCMC}=6.62\times 10^{-1}$; *SI Appendix*, Table S6).

Finally, we tested if the association between cooperation and relatedness might actually be owing to reverse causation, i.e., if cooperation drives relatedness. To do so, we included cooperation along with the ecological predictors in our model predicting within-host relatedness. We did not detect a significant effect for any of the six forms of cooperation (*SI Appendix*, Table S5) or a joint effect (Wald test on the posterior joint distribution of the six cooperative traits: $Chi^{2}=1.66$, df=6, p-value=0.95).

**Fig. 5.**
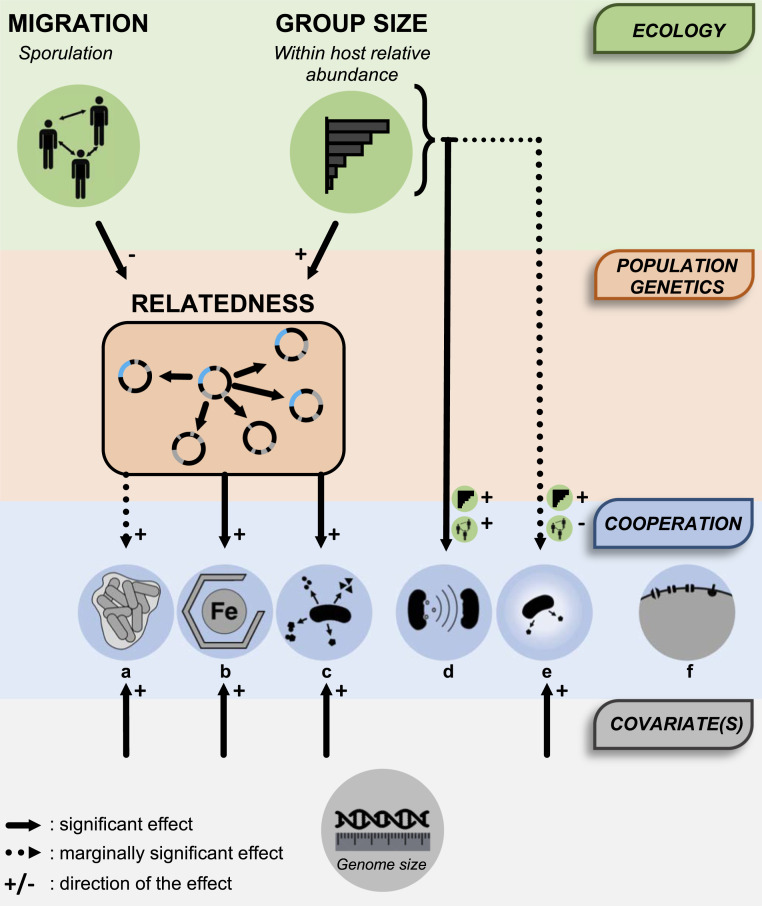
Kin selection explains the evolution of cooperation in the human gut microbiota. Summary schematic of the scenario supported by the path analysis conducted in this study is shown. Ecological factors (migration and group size) shape patterns of relatedness (average population genetic similarity), which, in turn, drive the evolution of cooperation. For certain specific forms of cooperation, ecological factors also have a direct effect: a, biofilm; b, siderophores; c, secreted products; d, quorum-sensing; e, antibiotic degradation; and f, secretion systems.

To summarize (Fig. 5), this path analysis shows that within the microbiome, migration and group size shape patterns of relatedness, which, in turn, drives the evolution of cooperation. Therefore, these ecological factors have an indirect effect on cooperation, via their effects on relatedness. Relatedness retains a direct positive effect on cooperation that is not accounted for by these ecological factors. Finally, ecological factors also have, particularly for group size, a direct effect on cooperation for some specific forms of cooperative behavior (Fig. 5).

## Discussion

### Relatedness in the Human Gut Microbiome.

Defining the “reference” and the “target” populations, respectively, implies a choice about the scale of competition (who do beneficiaries and altruists compete against?) and the scale of interaction (who are the potential beneficiaries?). Precise quantification of those scales, and the population genetic processes at work within the human gut microbiome, is a current avenue of research enabled by recent advances in strain-level resolution bioinformatics tools (54). Evidence of the worldwide spread of strains (46, 47) and host-strain replacement (48) suggests that the across-host population is the relevant scale of competition. Regarding the scale of interaction, using the host whole-gut population means that our estimate is a lower-bound estimate of relatedness. Indeed, while bacteria can potentially interact at the host scale (especially via their effects on the host), in some cases, there will be more within-host structure and localized interactions. In those cases, interacting individuals will be, on average, more similar than what we estimate from the whole gut, and true relatedness will be higher than that estimated from the whole gut. This lower-bound estimate remains predictive of the evolution of cooperation within the gut microbiota. This suggests that whole within-host scale interactions and global across-hosts scale of competition are an accurate depiction of the average population structure of human gut bacterial species.

### Relatedness Predicts Microbial Cooperation.

Our comparative analysis tests the relation between genomic relatedness and cooperative gene content over the full diversity of the human gut microbiota for six forms of cooperation. Various mechanisms that could invalidate Hamilton’s prediction have been highlighted by experimental and theoretical work. However, only a comparative approach allows us to actually test the general importance of those mechanisms in microbial evolution.

Some claims imply that that the inclusive-fitness theory is altogether unable to provide useful calculations for microbial evolution (25), arguing that it fails to take into account features that generally characterize the microbial world (36). Such an argument is directly contravened by the empirical evidence presented here. Relatedness does hold predictive power of the gut-microbe cooperative gene content across the full diversity of the human gut microbiota—that is, over a wide range of species ecology and life-history details.

Other arguments are focused on specific mechanisms that can lead to the break of Hamilton’s prediction, the question then being whether bacterial cooperative behaviors generally have these features or not. For example, benefits can be synergistic. Such an accelerating benefit curve implies a relation between group size and the benefit of cooperation: There must be enough cooperators for cooperation to pay (55). We find that group size has a significant predictive effect on number of quorum-sensing genes and a marginal effect on numbers of antibiotic-degrading enzymes, while for these two traits, relatedness does not have a predictive effect. This supports a scenario of synergistic benefits for these two traits and the claim that in such cases, group size prevails over relatedness at driving the evolution of public-goods production. Yet, synergistic benefits are known for siderophores as well (56, 57), for which we did find an effect of relatedness, but not of relative abundance. More generally, when looking at the overall effect of relative abundance, our meta-analysis model shows that it only has a marginal overall effect of relative abundance on cooperation, while relatedness holds an overall significant effect. Together, these results show that, while synergistic benefits may, in some cases, have a larger effect than relatedness, the general importance of this mechanism in the evolution of microbial cooperation remains limited and does not lessen the importance of relatedness. Similarly, the significant positive relation we observed between relatedness and siderophores suggests that the privatization of benefits via strain-specific receptors (34) does not disrupt Hamilton’s central prediction. Although such privatization may exist, the effect of relatedness remains dominant. Akin to the privatization of siderophores, it has been suggested that quorum-sensing acts as a mechanism of reciprocity and kin recognition (58, 59), in which case it does not require high relatedness for it to be favored. In the present results, the importance of this mechanism for quorum-sensing evolution cannot be distinguished from that of synergistic benefits.

Finally, cross-species benefits of public goods were shown to minimize the role of relatedness on the evolution of public-goods production (35), suggesting that Hamilton’s prediction may not hold in complex real-world MCs. Our results show that the existence of a highly connected metabolic network in the human gut microbiota (60, 61) does not curtail relatedness’ predictive power for the evolution of cooperative gene content across the gut-microbiota phylogeny. This suggests that the role of community context in the long-term evolution of cooperation does not overcome the importance of population-genetics context (relatedness), even in some highly complex and connected communities, such as the human gut microbiota. This may depend on the type of community (e.g., mammalian microbiome vs. MC community engineering). For example, manipulating cooperation to engineer successful human-gut fecal-transplant communities (62) may involve different challenges than manipulating cooperation for soil bio-remediation (63).

### Emphasizing Ecology vs. Relatedness.

Relatedness itself is likely a consequence of many aspects of organismal ecology, which create assortment (64). This motivates criticism of the emphasis on relatedness as an explanation for cooperation, while ecological factors and life-history traits are the ultimate drivers (25, 65). Indeed, our results depict a general scenario, where various ecological factors ultimately drive cooperation indirectly via their effects on relatedness: Although migration has no effect on cooperation, it does drive relatedness, and relatedness drives cooperation. Some ecological factors may act both indirectly and directly, as our results show for relative abundance, and the direct effects may even overcome that of relatedness in some cases. Yet, the predictive effect of relatedness on microbe cooperative gene content remained as strong after controlling for those two ecological factors. This means that relatedness is not captured by the combination of migration and group size. This gets to the heart of why relatedness is a relevant quantity for broad predictive ability: It is a population genetic parameter which collapses the complexity of various aspects of organismal ecology, while it remains poorly understood how a variety of ecological factors collectively shape relatedness, even less so at a broad taxonomic level. As such, relatedness provides a unifying variable (27) to make general predictions.

Here, our results show that the classic prediction of the infinite-island model that increased group size leads to reduced relatedness does not hold in microbes, at least in the context of the human gut microbiome. One possibility is that blooms (i.e., large increase in group size) of individual taxa in the microbiota are associated with genetic bottlenecks. This might be the case if between- and within-species competitive abilities are positively correlated—for example, if strains that reach high abundance provoke a host immune response which clears competitor strains. Therefore, rather than shifting our focus away from the influence of relatedness on cooperation (25), it is critical to understand how ecological factors shape it. The onset of tools and methods for tracking strains in metagenomic data (48, 66) is a promising avenue in which to do so.

## Conclusion

The ultimate test of any general evolutionary theory is in its ability to explain patterns of trait evolution across taxa. Overall, our results strongly support Hamilton’s central prediction that increased relatedness drives the evolution of cooperation. Our results hold across the human gut microbiota and reveal insights into the drivers of this key population genetic parameter. While debate continues over the exactness and assumptions of the inclusive fitness framework and kin-selection models, it is clear that the central insight of Hamilton’s theory is general and holds predictive power in complex real-world communities. Broad predictive ability is what is needed to turn microbiome research into a predictive science. Given the role of relatedness at driving microbial cooperation, the generally high measures of relatedness we observed in human gut microbes reinforce the idea that cooperation might be ubiquitous and play a key role in driving our gut MC dynamics (17). This is not only of importance for evolutionary biology, but also for medicine, as microbes can have their largest effects on health when they cooperate to help or harm their host (6, 15, 67). Hamiltonian medicine (62, 68)—the manipulation of relatedness in our microbes—may offer opportunities to steer microbial cooperation in ways to enhance human health.

## Materials and Methods

### Metagenomic Samples.

We used healthy individuals’ stool metagenomes from the Human Metagenome Project (HMP) (44) (HMP portal, accessed April 2020, under: Project > HMP, Body Site > feces, Studies > WGS-PP1, File Type > WGS raw sequences set, File format > FASTQ. The resulting manifest file is available at https://github.com/CamilleAnna/HamiltonRuleMicrobiome_gitRepos.git). For each host, we kept the earliest time point available and quality-filtered reads using the MOCAT pipeline with default settings (64). This resulted in 239 individual host metagenomic samples included in the analysis (list and access link provided in Dataset S1).

### Relatedness Calculation.

We used the strain-profiling pipeline MIDAS (49) to identify species in each metagenomic sample, estimate their relative abundance, and compute allele frequencies at each genomic site of the core genome. Specifically, we ran “run_midas.py snps” on the 239 metagenomes and identified 141 species meeting the default minimum coverage requirement for allele-frequency computation along the entire genome (per-host-per-species allele frequencies). We then used “merge_midas.py snps” to identify core genomic sites and compute diversity at those sites.

After quality filtering of samples and genomic sites (which we left to MIDAS defaults), core genomic sites were identified as sites present in most samples. For this, we set options *site_prev 0.90*, which means that a genomic site is considered part of the core genome if it is present in >90% of the samples. At this stage, we excluded *Bacteroides_xylanisolvens_57185*, which had a substantially smaller number of genomic sites passing quality filtering, resulting in a very small core genome size.

Finally, we used these diversity estimates to run “snp_diversity.py” to compute the within-sample (*sample_type per-sample*) and across-samples (*sample_type pooled-samples*) diversity. Computing relatedness requires at least two hosts, so we filtered out species present in only a single host. We then proceeded to computing relatedness on the remaining 101 species.

Following Lynch and Ritland (69), the genomic similarity averaged over any random pair of haploid individuals in a population is given by:

$$\bar{S}=\overset{n}{\underset{i=1}{\sum }}\overset{n}{\underset{j=1}{\sum }}p_{i}p_{j}S_{ij}$$ , [1]

where $p_{i}$ and $p_{j}$ denote the frequencies of haplotypes i and j and $S_{ij}$ the proportion of identical genomic sites for this interacting pair. This can also be derived from the allelic frequencies at each site n in a genome of length k. The average genomic similarity is:

$$\bar{S}=\frac{1}{k}\overset{k}{\underset{n=1}{\sum }}\overset{4}{\underset{a=1}{\sum }}p_{a}^{2},$$ [2]

with $p_{a}$ the frequency of allele a (out of the four possible alleles A, T, C, or G) at site k. This quantity is simply the average probability of being identical by state (or expected homozygosity in diploids) or 1− *genetic diversity*.

We used the MIDAS “diversity” function with default parameters to compute diversity within-hosts and diversity across-hosts to derive, respectively, the within-hosts (${\bar{S}}_{s,h}$) and across-hosts (${\bar{S}}_{s}$) average probability of being identical by state. ${\bar{S}}_{s,h}$ is the genomic similarity of species *s* within the subpopulation living in host h. ${\bar{S}}_{s}$ is the similarity that would be expected between any two random bacteria of that species’ entire population. The genetic relatedness for species s in host h is then:

$$r_{s,h}=\frac{{\bar{S}}_{s,h}-{\bar{S}}_{s}}{1-{\bar{S}}_{s}}.$$ [3]

Similar as an $F_{ST}$, an $r_{s,h}>0$ denotes a higher genomic similarity between any pair of interacting bacterium within a host gut subpopulation than would be expected from the average similarity in the global population, i.e., the statistical association between two interacting individuals relative to the average population, as defined by Hamilton’s kin-selection coefficient of relatedness (20).

### Secretome Size.

For each species, we downloaded the coding DNA sequence (CDS) fasta sequences from the PATRIC database (ref. (70); accessed January 29th, 2019). We used the same genomes as the reference genomes used for these species in the MIDAS database. We then ran PSORTb 3.0 (71) to determine protein localization. The secretome size is the number of CDS coding for a product predicted to have an extracellular final localization. PSORTb requires information on the gram profile. We assigned these following Bergey’s manual of systematic bacteriology (72) and/or the gram profile reported in the original descriptions of the species. The final secretome size obtained as well as the gram profiles are reported in Dataset S1.

### Cooperation Quantification from GOs.

We established a list of “social GO terms” corresponding to five well-described forms of bacterial social behaviors (biofilm formation, quorum-sensing, secretion systems, siderophores production and usage, and antibiotic degradation). To do so, we first identified a broad list of 14,702 “bacterial GO terms” by annotating all of the representative genomes cataloged in the MIDAS database (5,944 genomes) with GO terms using PANNZER2 (73) with default settings and keeping all hits (this full list is reported in Dataset S1). From this bacterial GO set, we identified a list of “bacterial cooperation GO terms” for the five types of cooperative behaviors. We first established a list of keywords describing those behaviors using the 10 most cited reviews on the topic (web of science search “TI=((microb* OR bacter* OR microorganis* OR micro-organis*) AND (coop* OR social*)”, selecting English, Reviews, All field, and manually filtering out reviews that were not specifically on microbial cooperation. The selected reviews are reported in Dataset S1). From the full list of bacterial GOs, we performed a keyword match to retrieve all GO terms containing these social keywords, as well as all corresponding child terms and direct parent term. This gave a list of 673 potentially social GO terms. Finally, we manually curated this list to ensure that the terms selected were specific enough. For example, while “polysaccharide production” could refer to a general aspect of metabolism, “extracellular polysaccharide production” can confidently be associated with biofilm formation. The detailed curation process is provided in Dataset S1. The final list of social GO terms comprises 118 terms (biofilm, 48; quorum sensing, 5; secretion systems, 11; siderophores, 29; and antibiotic degradation, 25; listed in Dataset S1). To quantify cooperation for these five classes of cooperative behaviors in our study species, we annotated their genome with GO using PANNZER2 with default settings and retaining only the top hit in each of the three ontologies (biological processes, cellular compartment, and molecular function). We then quantified cooperation for each of the five classes as the number of CDS for which at least one of its associated GO terms falls within the list of social GO terms for each given cooperation type. These counts are reported in Dataset S1.

### Phylogenetic Comparative Analyses.

For all comparative analyses, we built phylogenetic mixed models implemented in a Bayesian framework using the MCMCglmm package (74) in R (version 3.5.2) (75). To control for the phylogenetic relationships among species, we used the phylogeny provided in the MIDAS database, which we trimmed to keep our focus species-only and ultrametricized using chronopl function in ape (76). We ran all models for 1 million iterations with a burn-in phase of 5,000 and a thinning interval of 50. We used visual inspection of traces, as well as the Gelman–Rubin tests (77) on two independent chains to assess model convergence. Across all models and all effects estimated, the maximum potential scale-reduction factor observed was 1.03. The model summaries for the first chain of each model are provided in *SI Appendix*, Tables S1–S5. We report the significance of our fixed effects in terms of $P_{MCMC}$, which is twice the posterior probability that the estimate is negative.

### The Effect of Mean Relatedness on Cooperation.

We ran six models, one for each form of cooperation (secretome size and the five GO-based measures).). Each model was a univariate mixed model with a Poisson error structure, with cooperation (i.e., a number of genes) as the response variable:

$$\begin{array}{ll} E\left[Y_{i}\right] & =\beta_{0}^{Y}+\beta_{r}^{Y}R_{i}+\beta_{n}^{Y}log\left(N_{i}\right)+u_{p,i}^{Y}+\epsilon_{i}^{Y}\\ Y & ∼Pois\left(E\left[Y_{i}\right]\right), \end{array}$$ [4]

with $R_{i}$ denoting the mean relatedness of species i and $\beta_{r}^{Y}$ the regression coefficient of cooperation Y on relatedness. We also include $log\left(N_{i}\right)$ as predictor with $N_{i}$ the number of CDS not involved in the cooperative behavior Y (i.e., total number of CDS−Y), which we include in the model to account for potential nonlinear scaling of Y with genome size (*SI Appendix*, *SI Text*). The term $u_{p,i}^{Y}$ is the phylogenetic species effect on Y—that is, the amount of interspecies variance in Y explained by a Brownian motion model of evolution along the phylogeny. The residual variance $\epsilon_{i}^{Y}$ is equivalent to the nonphylogenetic interspecies variance in this case, as there is a single measure of mean relatedness per species. In the model for secretome size, we also included the gram profile in main effects to estimate a different intercept for each profile, since the PSORTb algorithm differs between Gram-positive and Gram-negative bacteria. We used an inverse-Wishart prior with expected variances set to one and degree of belief set to 0.002 for the residual variance in *Y* and for the phylogenetic random effects. For fixed effects, we used MCMCglmm’s default uninformative normally distributed prior with mean zero and variance of $10^{10}$. The model summaries are provided in *SI Appendix*, Table S1. We describe in *SI Appendix*, *SI Text* a bivariate formulation of these models, allowing us to account for uncertainty in the relatedness predictor. The summaries for these sets of models are provided in *SI Appendix*, Table S2.

In order to test if relatedness retains a significant explanatory power after controlling for relative abundance and migration, we added in this model the mean relative abundance $A_{i}$ and sporulation scores $S_{i}$ as fixed predictors:

$$\begin{array}{ll} E\left[Y_{i}\right] & =\beta_{0}^{Y}+\beta_{r}^{Y}R_{i}+\beta_{a}^{Y}A_{i}+\beta_{m}^{Y}S_{i}+\beta_{n}^{Y}log\left(N_{i}\right)+u_{p,i}^{Y}+\epsilon_{i}^{Y}\\ Y & ∼Pois\left(E\left[Y_{i}\right]\right), \end{array}$$ [5]

with $\beta_{a}^{Y}$ and $\beta_{m}^{Y}$ the regression coefficients of cooperation Y relative abundance and sporulation score, respectively. As before, we used an inverse-Wishart prior with expected variances set to one and degree of belief set to 0.002 for the residual variance in *Y* and the phylogenetic random effects. We used MCMCglmm’s default uninformative normally distributed prior with mean zero and variance of $10^{10}$ for all fixed effects. In all cases, the potential scale reduction factor was <1.01. The model summaries are provided in *SI Appendix*, Table S4.

### Drivers of Relatedness.

To assess the effect of ecological factors on relatedness, we constructed a Gaussian phylogenetic mixed model of relatedness with sporulation scores (i.e., migration) and relative abundance (i.e., group size) as fixed predictors:

$$\begin{array}{ll} E\left[R_{i}\right] & =\beta_{0}^{R}+\beta_{a}^{R}A_{i}+\beta_{m}^{R}M_{i}+u_{h}^{R}+u_{s}^{R}+u_{p}^{R}+\epsilon_{i}^{R}\\ R & ∼Gauss\left(E\left[R_{i}\right]\right), \end{array}$$ [6]

with $\beta_{a}^{R}$ and $\beta_{m}^{R}$, respectively, the regression coefficient of relatedness R on relative abundance A and sporulation score M. We partitioned the variance in relatedness into that which is explained by the Brownian motion model of evolution on the phylogeny, a species-specific component, and a host component, by treating those three sets of effects as random ($u_{h}^{R}$, $u_{s}^{R}$, and $u_{p}^{R}$). We used a default uninformative normally distributed prior with mean zero and variance of $10^{10}$ for both fixed effects and a parameter expanded prior for the three random effects (G1=G2=G3=list(V=1,nu=1,alpha.mu=0,alpha.V=1000)). We used an inverse-Wishart prior with expected variance set to one and degree of belief set to 0.002 for residual variance. The model summary is provided in *SI Appendix*, Table S3.

Finally, we added cooperation (for all six forms) as fixed predictors in this model to assess their effect on shaping relatedness as well. We assessed their significance both individually from each effect posterior distribution, as well as their collective effect using a Wald test [package aod v 1.3.1 (78)] on the variance–covariance matrix of the MCMCglmm model fit for the six fixed predictors corresponding to the six forms of cooperation. The model summary is provided in *SI Appendix*, Table S5.

### Meta-Analyses.

To quantify an overall slope of cooperation regressed on relatedness, we conducted a random-effects meta-analysis over the six phylogenetic mixed models. Each of the six classes of cooperation captures distinct cooperative behaviors (i.e., different sets of genes; *SI Appendix*, Fig. S6) and also differ by technical aspects (algorithmic vs. annotation-based). The global estimate obtained from the meta-analysis accounts for the uncertainty in each trait-specific estimate, arising from both this technical aspect and from the biological specificity of each form of cooperation.

We extracted the slope mean and SE directly from the phylogenetic mixed model posteriors. The analysis was implemented in a frequentist setting by using the R package metaphor (v 2.1-0.) (79) where the model is:

$$y_{i}∼\mu +m_{i}+\epsilon_{i}.$$ [7]

The Gaussian trait, y (estimate of the slope of cooperation over relatedness), of species i is given by the grand mean (μ) plus random effects due to measurement error ($m_{i}$) and residual ($\epsilon_{i}$). We seek to estimate the overall effect, μ. We conducted in total three meta-analyses: 1) over the six models with relatedness only as predictor; 2) over the six models with relatedness, relative abundance, and sporulation scores as predictors; and 3) over the models accounting for uncertainty in relatedness (see *SI Appendix*, *SI Text* for details). The model summaries are provided in *SI Appendix*, Table S6.

### Relative Abundance and Sporulation Scores.

We directly pulled species relative abundance from the MIDAS output (*Relatedness Calculation*). The abundance values are reported in Dataset S1. We computed sporulation scores for each of our focus species. Briefly, we retrieved the sequence of 66 characterized sporulation signature genes (80) from the National Center for Biotechnology Information and screened the genomes of our focal species for these sporulation genes. Following specifications from ref. 80, each sporulation signature gene was either considered present or absent in a genome based on blast identity, with an e-value cutoff of 1e-10. The copy number of a gene in a genome was not considered—e.g., if a gene was present twice in the genome, it was still counted the same as a gene present once. Each genome, therefore, has a maximum of 66 sporulation genes present (sporulation score of 1) or a minimum of no sporulation signature genes (sporulation score of 0). The computed scores are provided in Dataset S1.

### Supporting Information Appendix.

*SI Appendix* is provided for this manuscript, containing *SI Text*, Figs. S1–S6, Tables S1–S6, and legends for Dataset S1.

## Supplementary Material

Supplementary File

Supplementary File

## Data Availability

The metagenomic data are publicly available at the HMP portal (accessed April 2020, under: Project > HMP, Body Site > feces, Studies > WGS-PP1, File Type > WGS raw sequences set, File format > FASTQ). All codes (as well as the assembled data) are available in GitHub (https://github.com/CamilleAnna/HamiltonRuleMicrobiome_gitRepos.git). Dataset S1, analyses codes, and manifest files for the public metagenomes we used from the Human Microbiome Project portal are available in Zenodo (DOI: 10.5281/zenodo.4454867) (81). The assembled dataset for the phylogenetic comparative analysis is provided in Dataset S1.
