# Supplementary material for: Kin selection explains the evolution of cooperation in the gut microbiota
Source: Proc Natl Acad Sci U S A. 2021 Feb 1;118(6):e2016046118. doi: 10.1073/pnas.2016046118 (PMC8017935; doi:10.1073/pnas.2016046118)
Supplement: Supplementary File [file pnas.2016046118.sapp.pdf]

PNAS
www.pnas.org

Supplementary Information for

Kin selection explains the evolution of cooperation in the gut microbiota

Camille Simonet and Luke McNally

Corresponding Author name.
E-mail: camille.simonet@ed.ac.uk

This PDF file includes:

Supplementary text
Figs. S1 to S6
Tables S1 to S6
Legend for Dataset S1

Other supplementary materials for this manuscript include the following:

Dataset S1

Supporting Information Text

Microbial genomes build up in the kin selection framework

The purpose of this model is to better understand the dynamic of the process we hypothesised our data have been generated from, shaping the structure of our statistical modelling (particularly regarding genome size effect). We considered the build-up of a genome as a sampling process from a pool of available genes (the bacterial pan-genome). A species with a bigger genome may contain more cooperative genes solely because of this sampling process. To understand what the exact relationship between genome size and genome content in cooperative gene might be, one can model the build-up of the genome as follows. A genome contains a number H of housekeeping genes (i.e. essential genes necessary to perform the cell cycle) that are constant in time. In addition, it contains N non-social genes and S social genes that can be dynamically gained and lost. Those genes are sampled at rate β. Social genes are retained with a probability $p_s(r)$ which depends on the relatedness r. In contrast, the probability to retain a non-social gene depends on a probability p_s which does not depend on relatedness. For this heuristic model, we do not make any assumption about the shape of these two probability functions. Both social and non-social genes are lost at a rate α. The dynamics for the number of social and non-social genes is described by:

$$\frac{d_N}{d_t} = \beta p_n - \alpha(N - H)$$
$$\frac{d_S}{d_t} = \beta p_s(r) - \alpha S$$

The equilibrium number of non-social and social genes are therefore:

$$N^* = \frac{H\alpha + \beta p_n}{\alpha} = H + \gamma p_n$$
$$S^* = \frac{\beta p_s(r)}{alpha} = \gamma p_s(r)$$

With $\gamma = \frac{\beta}{\alpha}$ the gain to loss rate ratio. To model a discrete count response variable, the classic approach is to use a Poisson general linear model with a log link. The linear model therefore describes the log of the response variable, here, the log of the number of genes involved in cooperation, $log(S)$. At equilibrium, we have:

$$\begin{aligned} log(S) &= log(\gamma p_s(r)) \\ &= log(\gamma) + log(p_s(r)) \\ &= \frac{log(N-H)}{p_n} + log(p_s(r)) \end{aligned}$$

Which we can also write:

$$log(\frac{e^{log(N)} - H}{p_n}) + log(p_s(r))$$

We can now derive this expression of $log(S)$ relative to $log(N)$ to ask how does the log of the number of cooperative gene scale with the log of the number of non-cooperative genes:

$$\frac{d_{log(S))}}{d_{log(N))}} = \frac{n}{n-h}$$

Therefore, regardless of the shape of $p_s(r)$ we expect a super-linear scaling between the response and the predictor, on the link scale of a Poisson response model (that is, $log(S)$ for the response, and $log(N)$ for the predictor), simply as a result of the gene sampling process.

Accounting for uncertainty in relatedness estimates

In the main model, we used mean relatedness as predictor in the comparative analysis. There are actually several point estimates of relatedness: one per hosts within which a species was found. This means that relatedness was estimated in each species, and so with different levels of uncertainty because each species is found in a different number of host (2 to 227). When the predictor is measured with error, this can lead to bias in the estimate of true effect. We accounted for this in a more complex version of the model by explicitly modelling relatedness as:

$$R_i = \beta_0^{(R)} + u_{s,i}^{(R)} + u_{p,i}^{(R)} + \epsilon_i^{(R)}$$

Where $u_s^{(R)}$ and $u_p^{(R)}$ are respectively the vectors of non-phylogenetic and phylogenetic species effects on relatedness (superscript R). These two terms describe respectively the inter-species variance in relatedness explained by Brownian motion model of evolution and a species-specific component independent of the phylogeny, but is not attributable to measurement error. Unlike for relatedness, there is a single measure of secretome size per species, thus residual and species level variance are the same thing for secretome size. Therefore, the covariance between the species level variance for relatedness and residual

variance for secretome size ($\epsilon^{(Y)}$) measures how much correlation there is between secretome size and relatedness that doesn't conform to Brownian motion, and the covariance between inter-species phylogenetic variance for relatedness and inter-species phylogenetic variance for Y ($u_p^{(Y)}$) measures how much correlation there is that is explained by a Brownian motion model of evolution along the phylogeny. With a bivariate model formulation, we can then model cooperation as:

$$E[Y_i] = \beta_0^{(Y)} + \beta_n^{(Y)} log(N_i) + \beta_s^{(Y)} u_{s,i}^{(R)} + \beta_p^{(Y)} u_{p,i}^{(R)} + u_{p,i}^{(Y)} + \epsilon^{(Y)}$$
$$Y \sim Pois(E[Y_i])$$

N_i is the number of CDS not involved in the cooperative behaviour Y (i.e. total number of $CDS - Y$) which we include in the model to account for potential non-linear scaling of Y with genome size. The regression coefficient $\beta_s^{(Y)} = \frac{cov(\epsilon^{(Y)}, u_s^{(R)})}{var(\epsilon^{(Y)})}$ is the non-phylogenetic regression coefficient of the cooperative behaviour Y on relatedness, while $\beta_p^{(Y)} = \frac{cov(u_p^{(Y)}, u_p^{(R)})}{var(u_p^{(Y)})}$ is the phylogenetic regression coefficient of the cooperative behaviour Y on relatedness. The total regression coefficient of Y on relatedness can finally be computed from these two estimated (co)variance matrices:

$$\beta_R^{(Y)} = \frac{cov(u_p^{(Y)}, u_p^{(R)}) + cov(\epsilon^{(Y)}, u_s^{(R)})}{var(u_p^{(Y)}) + var(\epsilon^{(Y)})}$$

We implemented this model in MCMCglmm, using a bivariate model formulation to estimate the two aforementioned variance-covariances matrices. The phylogenetic covariance matrix between relatedness and secretome size was modelled between the ID random effect of both measures (`random =  us(at.level(trait,1)+at.level(trait,2)):species`). We used a parameter expanded algorithm to fit this structure, specifying the prior as a 2-dimensional scaled (by a 1000) F distribution with 1 degree of freedom in both the numerator and denominator (`G=list(G1=list(V=diag(2), nu=2, alpha.mu=c(0,0), alpha.V=diag(2)*1000))`). For the non-phylogenetic covariance matrix, it is a random effect – residual structure that we are fitting, that is, we model the covariance between the ID random effect for the relatedness (repeat measure trait) and the residual for cooperation (single-measure trait). We specified this by setting `covu = TRUE` in the prior specification for the residuals structure. This allows the residuals for this structure (`rcov = idh(at.level(trait,1)):species.ide`) to covary with the random effects specified by the final random effect structure (we added `idh(at.level(trait,2)):species.ide` to the random argument formula. With this model formulation, the residual prior specification is therefore a 2-dimensional matrix. We used a 2-dimensional inverse-Wishart distribution with expected (co)variances set to 1 and degree of belief set to 0.002 (`R1=list(V=diag(2), nu=0.002, covu=TRUE`). Finally the residual variance for relatedness (measurement error) was explicitly modelled adding `idh(at.level(trait,2)):units` to the `rcov` argument formula. For this we specified an inverse-Wishart prior with expected variances set to 1 and degree of belief set to 0.002 (`R2=list(V=1, nu=0.002)`). For fixed effects we used MCMCglmm's default uninformative normally distributed prior with mean 0 and variance of 10^{10}. In the model for secretome size, we also included the gram profile in the main effects to estimate a different intercept for each gram type, since the Psortb algorithm differs between gram-positive and gram-negative bacteria. We ran all models for 650,000 iterations with a burn-in phase of 150,000 and a thinning interval of 50. We used visual inspection of traces to assess model convergence. Model summaries are provided in supplementary table S2. We conducted a random effects meta-analysis on these models output exactly as described in the main text methods. The corresponding summary is provided in supplementary table S5.

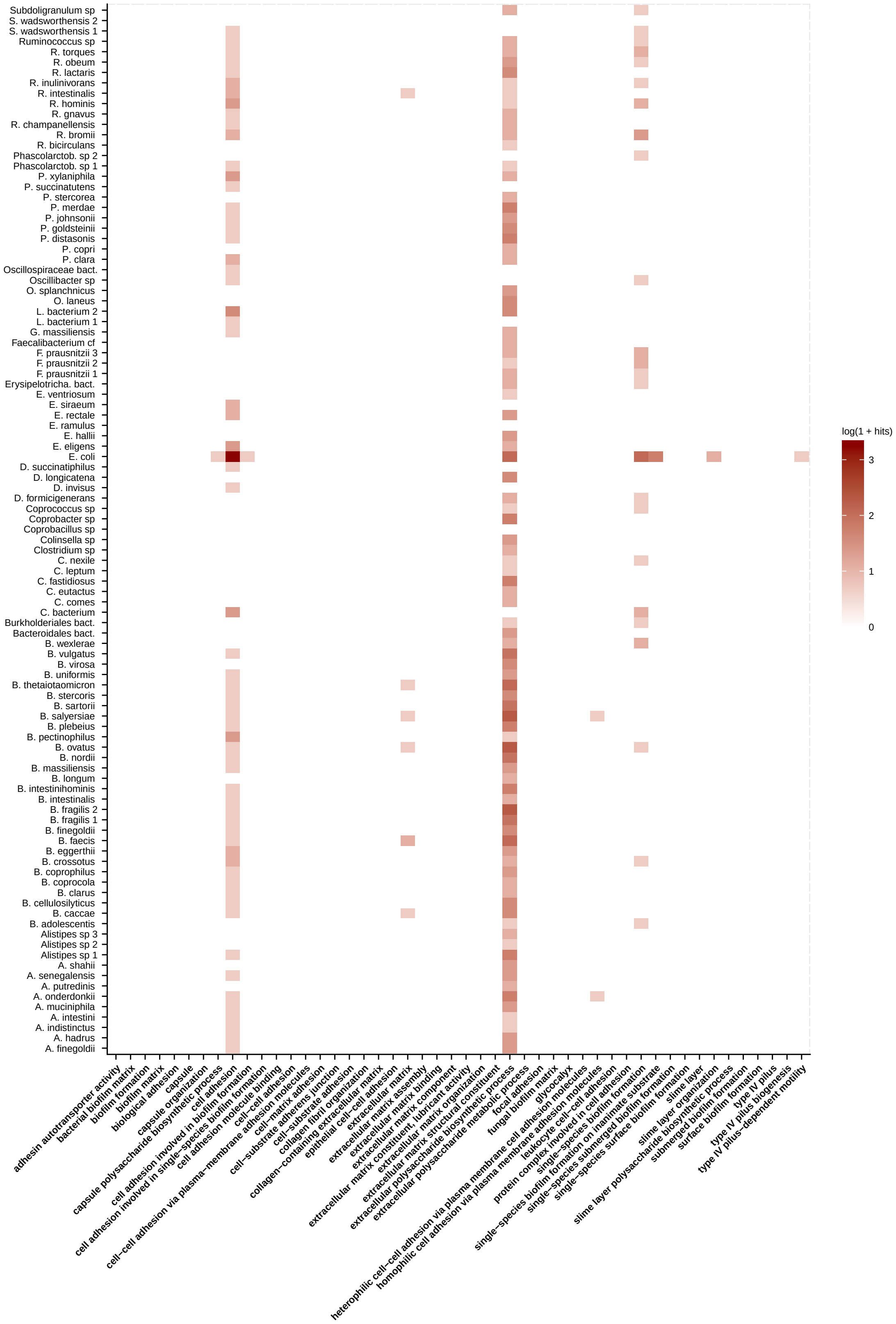

Fig. S1. GO terms contribution to the biofilm cooperation class. 42 GO terms were retained to capture bacterial cooperation in the form of biofilm formation. The heatmap shows, for each species, the number of CDS annotated with a GO matching one of these terms.

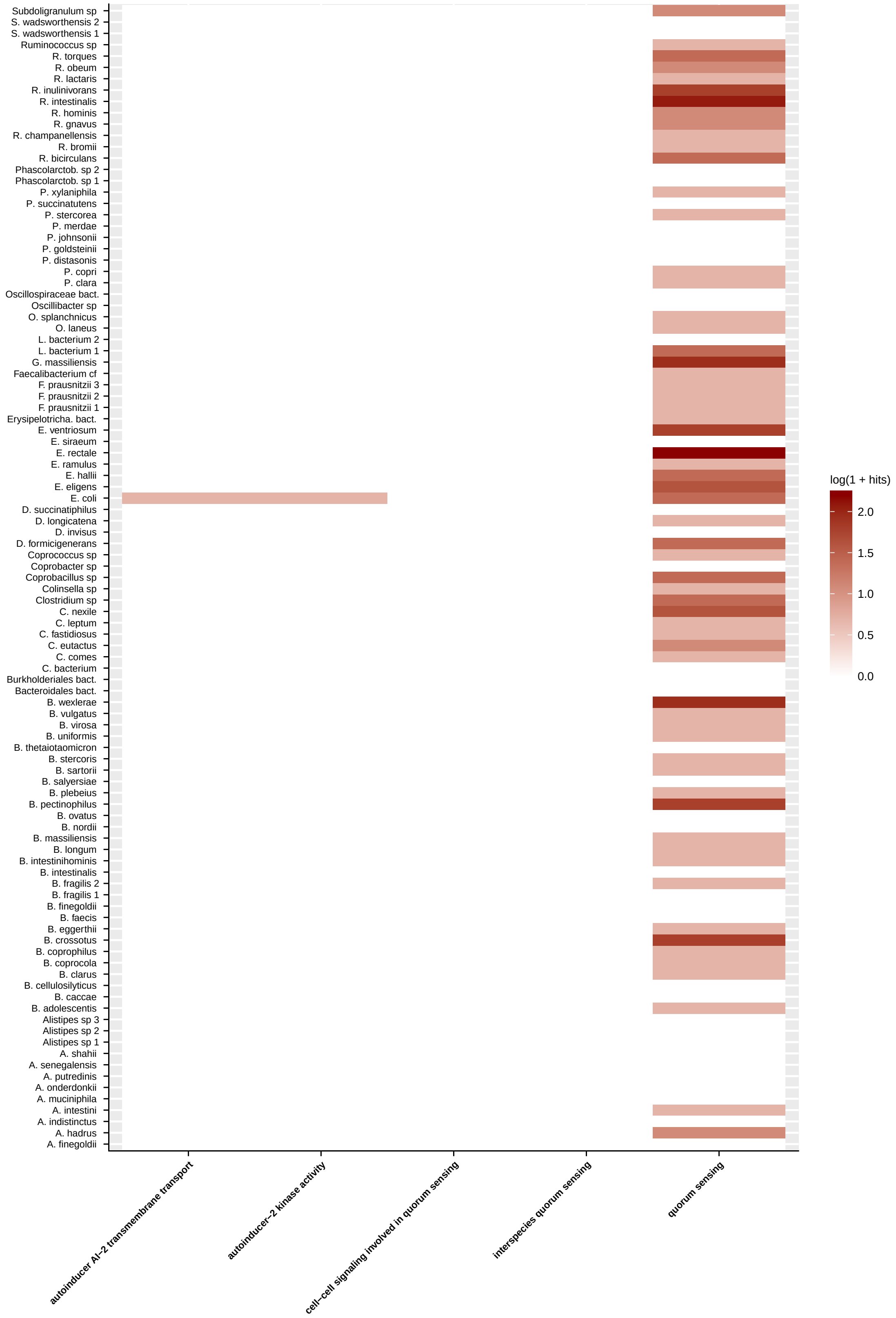

Fig. S2. GO terms contribution to the quorum-sensing cooperation class. 5 GO terms were retained to capture bacterial cooperation in the form of quorum-sensing. The heatmap shows, for each species, the number of CDS annotated with a GO matching one of these terms.

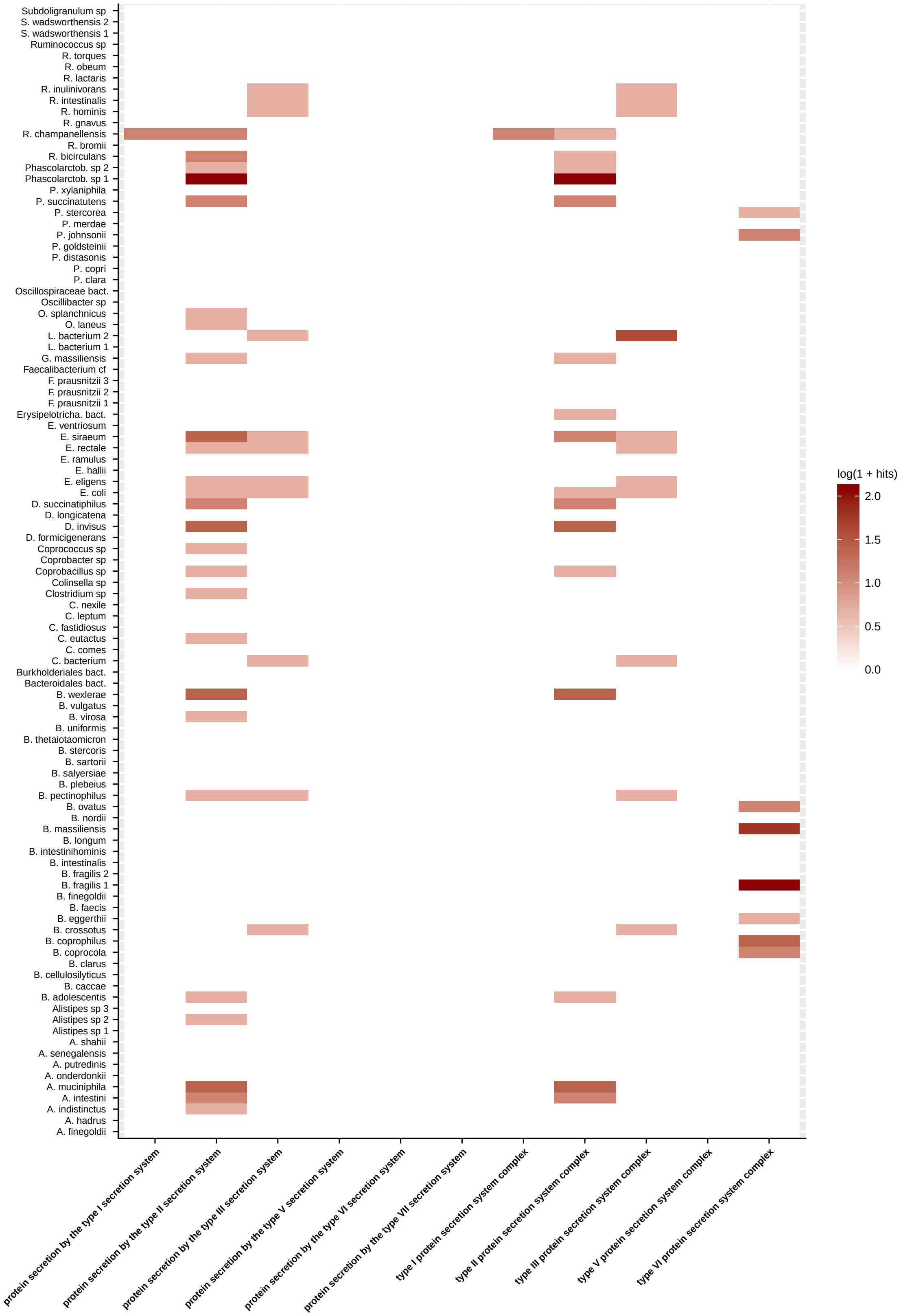

Fig. S3. GO terms contribution to the secretion systems cooperation class. 11 GO terms were retained to capture bacterial cooperation in the form of secretion systems. The heatmap shows, for each species, the number of CDS annotated with a GO matching one of these terms.

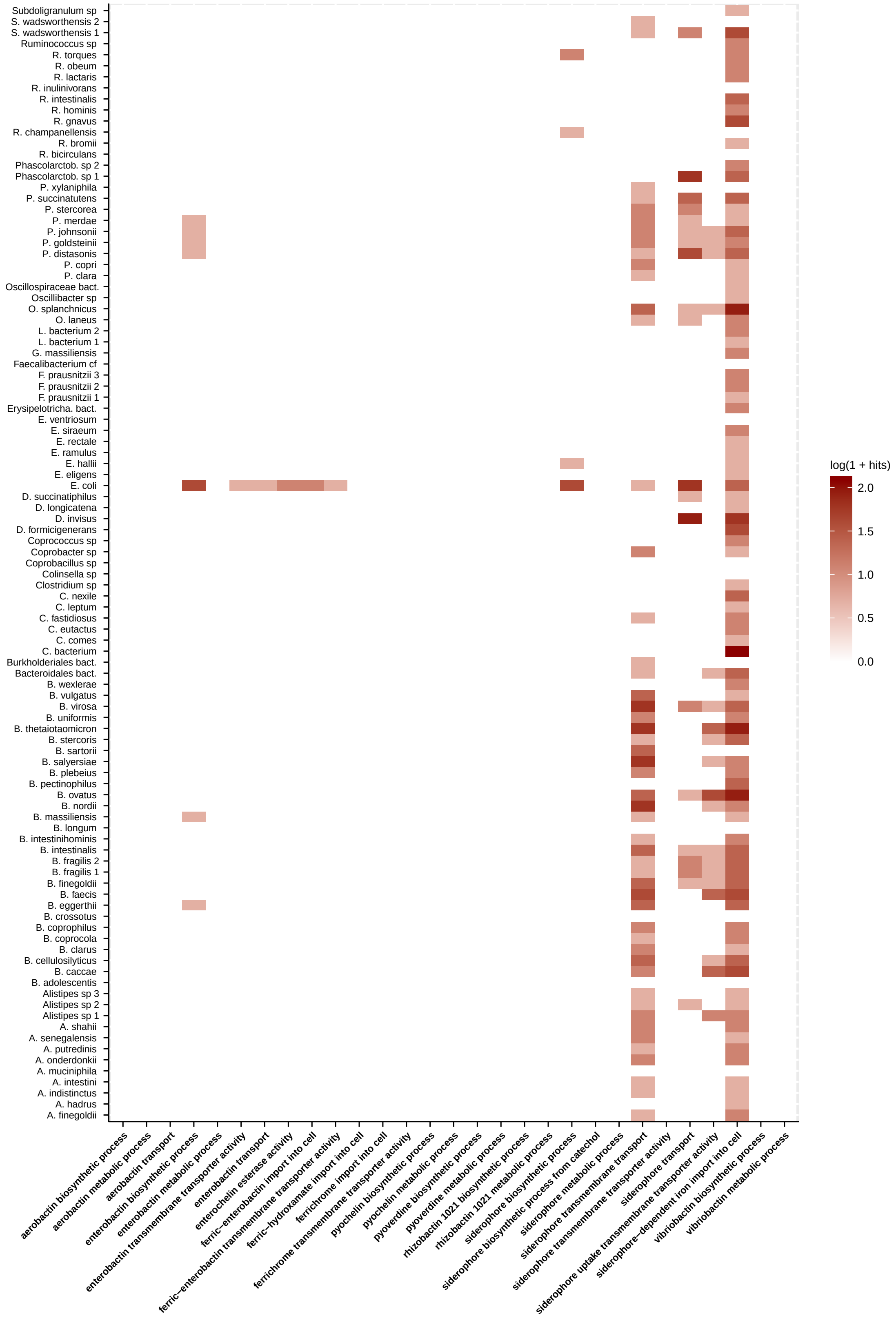

Fig. S4. GO terms contribution to the siderophores cooperation class. 29 GO terms were retained to capture bacterial cooperation in the form of siderophores production and usage. The heatmap shows, for each species, the number of CDS annotated with a GO matching one of these terms.

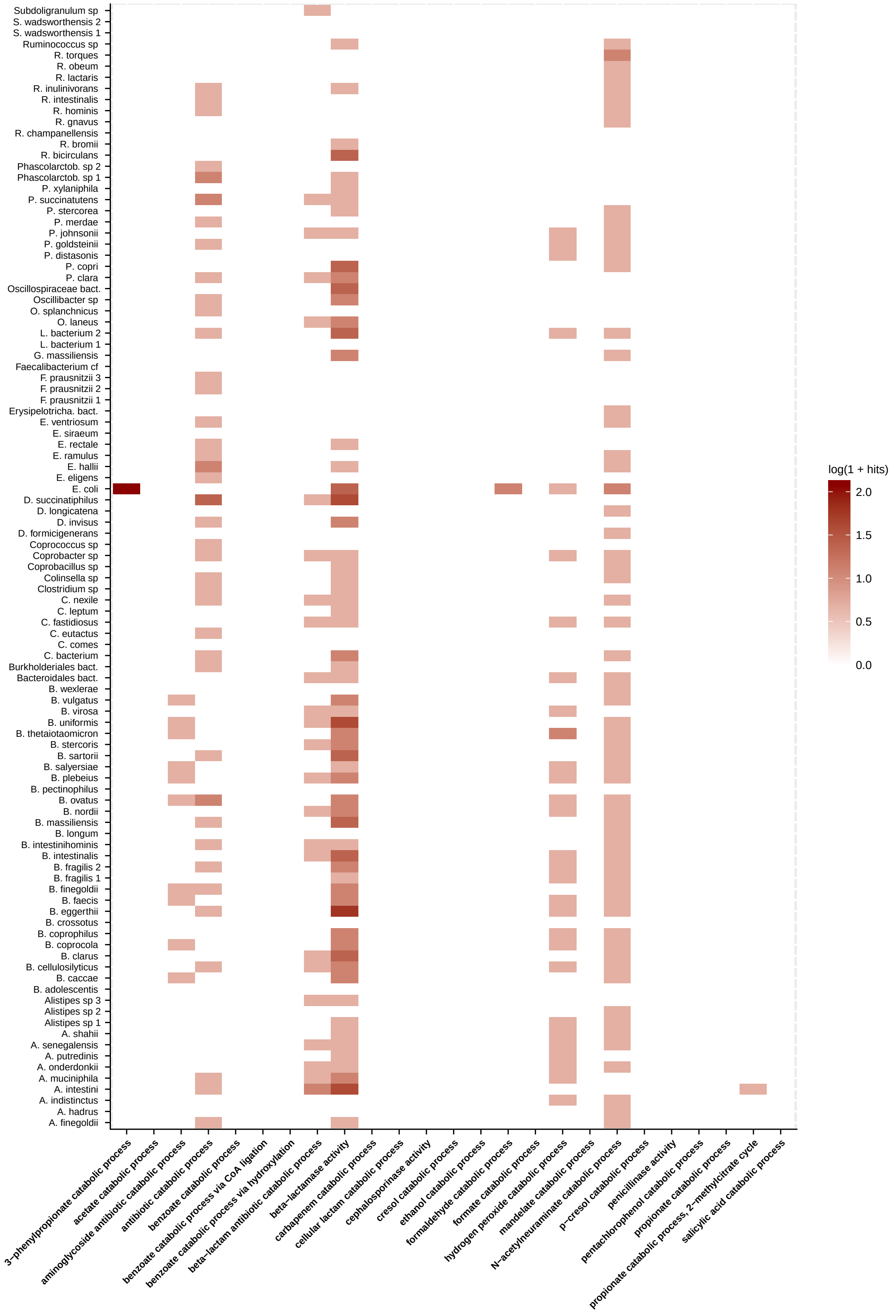

Fig. S5. GO terms contribution to the antibiotic degradation cooperation class. 25 GO terms were retained to capture bacterial cooperation in the form of antibiotic degradation molecules production. The heatmap shows, for each species, the number of CDS annotated with a GO matching one of these terms.

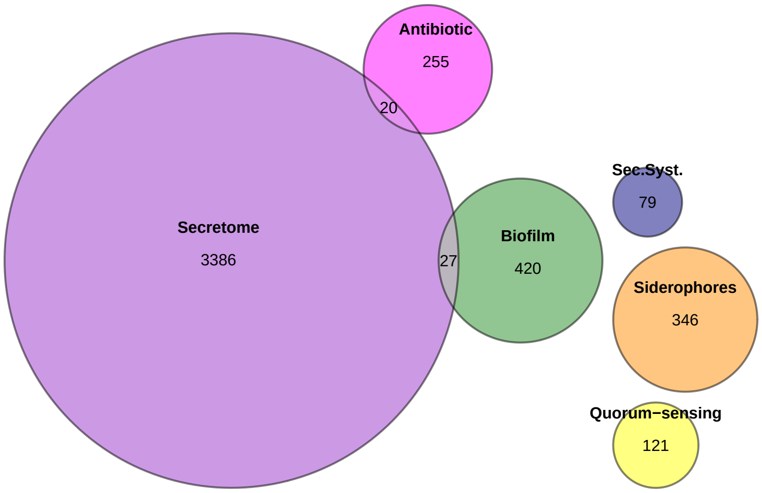

Fig. S6. Cooperative genes for 6 forms of bacterial cooperation. Across the 101 species, a total of 4607 genes were annotated as coding for a cooperative product. The Venn diagram shows the number of genes (across all species) falling within either of the 6 cooperation categories. A species-specific version of this figure is available in our repository at https://github.com/CamilleAnna/HamiltonRuleMicrobiome_gitRepos.git

Table S1. Model summaries for the phylogenetic mixed models of cooperation

Model	Structure	Effect	Posterior mean	CI95% lower	CI95% upper	Effective sampling	pMCMC
**Siderophores**							
	Fixed effect	Intercept	-10.572	-16.188	-5.198	3528	2.22e-04
		Mean relatedness	1.558	0.418	2.776	2548	9.56e-03
		Log(genome size)	1.322	0.699	1.973	3713	1.11e-04
	(Co)variance	Phylogenetic	0.488	0.207	0.850	2013	
		Residual (non-phylogenetic)	0.011	0.000	0.040	5921	
**Biofilm**							
	Fixed effect	Intercept	-9.275	-14.057	-4.389	3820	2.22e-04
		Mean relatedness	1.059	0.055	2.104	3029	0.046
		Log(genome size)	1.245	0.700	1.799	4084	1.11e-04
	(Co)variance	Phylogenetic	0.290	0.111	0.502	3335	
		Residual (non-phylogenetic)	0.012	0.000	0.043	3910	
**Antibiotic degradation**							
	Fixed effect	Intercept	-9.267	-14.409	-3.854	2552	1.56e-03
		Mean relatedness	0.861	-0.257	2.033	1933	0.142
		Log(genome size)	1.201	0.597	1.800	2764	4.44e-04
	(Co)variance	Phylogenetic	0.267	0.066	0.518	1558	
		Residual (non-phylogenetic)	0.009	0.000	0.034	5212	
**Secretome**							
	Fixed effect	Intercept	-7.681	-10.274	-5.116	8656	1.11e-04
		Mean relatedness	0.593	0.075	1.071	8494	0.019
		Log(genome size)	1.289	0.978	1.592	8715	1.11e-04
		Gram positive	0.602	0.372	0.850	8591	1.11e-04
	(Co)variance	Phylogenetic	0.134	0.051	0.220	6475	
		Residual (non-phylogenetic)	0.008	0.000	0.026	5899	
**Secretion systems**							
	Fixed effect	Intercept	-10.213	-25.456	3.635	3083	0.167
		Mean relatedness	0.479	-2.275	3.081	3637	0.723
		Log(genome size)	1.156	-0.467	2.881	3091	0.177
	(Co)variance	Phylogenetic	2.702	0.000	5.585	1384	
		Residual (non-phylogenetic)	0.378	0.000	1.331	1603	
**Quorum sensing**							
	Fixed effect	Intercept	-0.967	-10.983	9.199	1494	0.838
		Mean relatedness	0.393	-1.343	2.210	1748	0.669
		Log(genome size)	0.056	-1.125	1.238	1492	0.906
	(Co)variance	Phylogenetic	1.188	0.445	2.223	479	
		Residual (non-phylogenetic)	0.023	0.000	0.089	4413	

CI95%: 95% credible interval of the posterior distribution
pMCMC: taken as twice the posterior probability that the estimate is negative

Table S2. Model summaries for phylogenetic mixed models of cooperation, for the six forms of cooperation, when accounting for uncertainty in relatedness estimates. The total regression coefficient of the response trait over relatedness is the sum of the phylogenetic and non-phylogenetic (residual) covariances divided by the sum of the phylogenetic and non-phylogenetic (residual) variances

Model	Structure	Effect	Post. mean	CI95% lower	CI95% upper	Eff. samp.	pMCMC
**Siderophores**							
	Fixed effects	Intercept relatedness	0.741	0.608	0.884	9466	1.00e-04
		Intercept trait	-8.429	-13.598	-3.455	2324	1.80e-03
		Log(genome size)	1.196	0.573	1.834	2524	1.00e-04
	Phyl. (co)-variances	Relatedness	0.022	0.000	0.048	1549	
		Relatedness,Trait	0.035	-0.027	0.105	2218	0.2632
		Trait	0.613	0.250	1.030	1116	
	Species (co)-variances	Relatedness	0.009	0.001	0.017	1550	
		Relatedness,Trait	0.008	-0.008	0.029	1797	0.3666
		Trait	0.025	0.000	0.085	2073	
	Residual variance	Relatedness	0.044	0.042	0.046	10000	
**Biofilm**							
	Fixed effects	Intercept relatedness	0.761	0.625	0.916	7157	1.00e-04
		Intercept trait	-8.247	-12.651	-4.088	1008	4.00e-04
		Log(genome size)	1.216	0.711	1.772	1016	1.00e-04
	Phyl. (co)-variances	Relatedness	0.022	0.000	0.045	2051	
		Relatedness,Trait	0.050	-0.007	0.109	1453	0.065
		Trait	0.383	0.168	0.624	1051	
	Species (co)-variances	Relatedness	0.008	0.002	0.015	2516	
		Relatedness,Trait	0.004	-0.010	0.021	1328	0.6574
		Trait	0.014	0.000	0.048	1841	
	Residual variance	Relatedness	0.044	0.042	0.046	10000	
**Antibiotic degradation**							
	Fixed effects	Intercept relatedness	0.748	0.614	0.889	9248	1.00e-04
		Intercept trait	-8.004	-12.501	-3.591	1636	1.00e-04
		Log(genome size)	1.123	0.571	1.676	1662	1.00e-04
	Phyl. (co)-variances	Relatedness	0.020	0.000	0.045	1414	
		Relatedness,Trait	0.029	-0.015	0.084	1661	0.1948
		Trait	0.328	0.103	0.598	660	
	Species (co)-variances	Relatedness	0.009	0.002	0.017	1729	
		Relatedness,Trait	0.002	-0.013	0.017	1795	0.835
		Trait	0.012	0.000	0.037	2647	
	Residual variance	Relatedness	0.044	0.042	0.046	10624	
**Secretome**							
	Fixed effects	Intercept relatedness	0.738	0.596	0.869	10000	1.00e-04
		Intercept trait	-6.962	-9.464	-4.462	9068	1.00e-04
		Gram profile (negative)	-0.618	-0.837	-0.370	7537	1.00e-04
		Log(genome size)	1.332	1.014	1.644	9096	1.00e-04
	Phyl. (co)-variances	Relatedness	0.021	0.000	0.039	2655	
		Relatedness,Trait	0.031	-0.001	0.061	4067	0.0424
		Trait	0.165	0.081	0.265	5051	
	Species (co)-variances	Relatedness	0.006	0.001	0.012	2985	
		Relatedness,Trait	0.001	-0.006	0.009	3051	0.815
		Trait	0.007	0.000	0.022	4439	
	Residual variance	Relatedness	0.043	0.041	0.044	10000	
**Secretion systems**							
	Fixed effects	Intercept relatedness	0.743	0.609	0.889	9252	1.00e-04
		Intercept trait	-11.174	-24.455	1.162	1705	0.083
		Log(genome size)	1.326	-0.265	2.919	1749	0.098
	Phyl. (co)-variances	Relatedness	0.022	0.000	0.046	2085	
		Relatedness,Trait	0.089	-0.055	0.243	1982	0.209
		Trait	3.250	0.763	6.367	552	
	Species (co)-variances	Relatedness	0.008	0.001	0.015	2345	
		Relatedness,Trait	-0.014	-0.066	0.032	1567	1.4814
		Trait	0.247	0.000	0.910	1032	

Table S2. Model summaries for phylogenetic mixed models of cooperation, for the six forms of cooperation, when accounting for uncertainty in relatedness estimates. The total regression coefficient of the response trait over relatedness is the sum of the phylogenetic and non-phylogenetic (residual) covariances divided by the sum of the phylogenetic and non-phylogenetic (residual) variances *(continued)*

Model	Structure	Effect	Post. mean	CI95% lower	CI95% upper	Eff. samp.	pMCMC
	Residual variance	Relatedness	0.044	0.042	0.046	10000	
**Quorum sensing**							
	Fixed effects	Intercept relatedness	0.736	0.600	0.882	10000	1.00e-04
		Intercept trait	0.008	-8.540	8.687	924	0.997
		Log(genome size)	-0.026	-1.112	1.041	926	0.959
	Phyl. (co)-variances	Relatedness	0.021	0.000	0.047	1441	
		Relatedness,Trait	0.002	-0.077	0.092	1584	1.0294
		Trait	1.209	0.425	2.117	287	
	Species (co)-variances	Relatedness	0.009	0.002	0.017	1532	
		Relatedness,Trait	0.002	-0.019	0.025	1707	0.862
		Trait	0.025	0.000	0.091	1999	
	Residual variance	Relatedness	0.044	0.042	0.046	9689	

CI95%: 95% credible interval of the posterior distribution

pMCMC: taken as twice the posterior probability that the estimate is negative

Table S3. Model summary for the phylogenetic mixed model of relatedness (drivers of relatedness)

Structure	Effect	Posterior mean	CI95% lower	CI95% upper	Effective sampling	pMCMC
Fixed effects	Intercept	0.891	0.676	1.106	19007	5.00e-05
	Sporulation score	-0.692	-1.254	-0.100	16046	0.015
	Within host relative abundance	0.091	0.008	0.169	20000	0.025
Variances	Species, non-phylogenetic	0.007	0.001	0.015	3519	
	Species, phylogenetic	0.031	0.000	0.057	2875	
	Host	0.021	0.017	0.025	18904	
	Residual	0.026	0.025	0.028	20000	

CI95%: 95% credible interval of the posterior distribution
pMCMC: taken as twice the posterior probability that the estimate is negative

Table S4. Model summaries for the phylogenetic mixed models of cooperation when including sporulation scores and relative abundance as predictors

Model	Structure	Effect	Posterior mean	CI95% lower	CI95% upper	Effective sampling	pMCMC
**Siderophores**							
	Fixed effect	Intercept	-10.725	-16.228	-5.012	3400	2.22e-04
		Mean relatedness	1.432	0.243	2.679	2647	0.020
		Mean relative abundance	0.415	-3.378	4.191	3625	0.809
		Sporulation score	-1.600	-4.335	1.213	3547	0.261
		Log(genome size)	1.401	0.780	2.071	3798	1.11e-04
	(Co)variance	Phylogenetic	0.456	0.158	0.804	1520	
		Residual (non-phylogenetic)	0.011	0.000	0.040	5669	
**Biofilm**							
	Fixed effect	Intercept	-9.619	-14.447	-4.911	2539	1.11e-04
		Mean relatedness	1.015	-0.006	2.011	1715	0.057
		Mean relative abundance	2.227	-1.026	5.274	3203	0.172
		Sporulation score	-0.306	-2.589	1.703	3188	0.767
		Log(genome size)	1.289	0.745	1.827	2920	1.11e-04
	(Co)variance	Phylogenetic	0.265	0.084	0.496	1689	
		Residual (non-phylogenetic)	0.012	0.000	0.043	2779	
**Antibiotic degradation**							
	Fixed effect	Intercept	-9.332	-14.036	-4.591	2936	1.11e-04
		Mean relatedness	0.612	-0.465	1.660	2247	0.266
		Mean relative abundance	3.209	-0.008	6.256	3378	0.059
		Sporulation score	-1.795	-3.839	0.089	3455	0.067
		Log(genome size)	1.267	0.715	1.800	3174	1.11e-04
	(Co)variance	Phylogenetic	0.158	0.000	0.362	1143	
		Residual (non-phylogenetic)	0.010	0.000	0.036	5135	
**Secretome**							
	Fixed effect	Intercept	-7.586	-10.283	-4.915	8616	1.11e-04
		Mean relatedness	0.599	0.087	1.145	8519	0.030
		Mean relative abundance	-0.333	-2.038	1.398	8299	0.704
		Sporulation score	0.007	-1.336	1.347	9000	0.988
		Log(genome size)	1.280	0.954	1.605	8644	1.11e-04
		Gram positive	0.597	0.348	0.847	9000	1.11e-04
	(Co)variance	Phylogenetic	0.144	0.059	0.239	7773	
		Residual (non-phylogenetic)	0.008	0.000	0.026	6230	
**Secretion systems**							
	Fixed effect	Intercept	-11.008	-26.802	3.374	3702	0.145
		Mean relatedness	0.278	-2.437	3.236	3510	0.842
		Mean relative abundance	2.715	-8.050	13.282	3669	0.600
		Sporulation score	-1.370	-8.529	5.045	4559	0.690
		Log(genome size)	1.297	-0.516	3.054	3672	0.141
	(Co)variance	Phylogenetic	3.130	0.001	6.330	1423	
		Residual (non-phylogenetic)	0.344	0.000	1.270	2375	
**Quorum sensing**							
	Fixed effect	Intercept	-1.800	-10.666	7.690	1930	0.691
		Mean relatedness	0.644	-1.048	2.259	2132	0.454
		Mean relative abundance	7.386	1.229	13.246	2254	0.022
		Sporulation score	4.422	0.498	7.998	2858	0.026
		Log(genome size)	-0.048	-1.114	1.049	2090	0.936
	(Co)variance	Phylogenetic	0.764	0.149	1.559	813	
		Residual (non-phylogenetic)	0.028	0.000	0.112	4012	

CI95%: 95% credible interval of the posterior distribution
pMCMC: taken as twice the posterior probability that the estimate is negative

Table S5. Model summary for the phylogenetic mixed model of relatedness (drivers of relatedness) with cooperation (all six forms) included as fixed predictors

Structure	Effect	Posterior mean	CI95% lower	CI95% upper	Effective sampling	pMCMC
Fixed effects	Intercept	0.875	0.625	1.106	20000	5.00e-05
	Sporulation score	-0.648	-1.248	-0.068	20000	0.032
	Within host relative abundance	0.094	0.016	0.173	20000	0.016
	Biofilm	0.001	-0.010	0.011	19514	0.911
	Antibiotic degradation	-0.009	-0.031	0.012	19188	0.405
	Quorum sensing	-0.002	-0.026	0.021	20000	0.852
	Siderophores	0.006	-0.007	0.020	20000	0.357
	Secretion systems	-0.002	-0.022	0.017	19270	0.820
	Secretome	-0.000	-0.002	0.002	19517	0.876
Variances	Species, non-phylogenetic	0.004	0.000	0.009	7996	
	Species, phylogenetic	0.034	0.014	0.057	6182	
	Host	0.020	0.016	0.024	20000	
	Residual	0.025	0.024	0.027	20000	

CI95%: 95% credible interval of the posterior distribution

pMCMC: taken as twice the posterior probability that the estimate is negative

Table S6. Meta-analysis model summaries

Predictor	Estimate	Std. Err.	CI95% lower	CI95% upper	z value	p value
**Model 1**						
Mean relatedness	0.776	0.194	0.395	1.157	3.992	6.566e-05
Log(genome size)	1.227	0.113	1.005	1.449	10.837	2.304e-27
**Model 2**						
Within host relatedness	0.061	0.025	0.011	0.11	2.386	0.017
Log(genome size)	1.206	0.113	0.985	1.427	10.701	1.009e-26
**Model 3**						
Mean relatedness	0.743	0.198	0.354	1.131	3.748	1.779e-04
Mean relative abundance	1.786	1.019	-0.211	3.783	1.753	0.08
Sporulation score	-0.302	0.692	-1.659	1.054	-0.437	0.662
Log(genome size)	1.235	0.115	1.011	1.46	10.778	4.389e-27

CI95%: 95% confidence intervals

pMCMC: taken as twice the posterior probability that the estimate is negative

Model 1: meta-analysis over the models of cooperation with mean relatedness as predictor

Model 2: meta-analysis over the models of cooperation accounting for uncertainty in relatedness estimates

Model 3: meta-analysis over the models of cooperation with mean relatedness and sporulation scores and relative abundance as predictors

SI Dataset S1 (SI_dataset.xlsx)

Excel spreadsheet containing:

- SI1: Metagenomic samples used and access links.
- SI2: Reference on bacterial cooperation retreived from Web of Science search: TI¯((microb* OR bacter* OR microorganis* OR micro-organis*) AND (coop* OR social*)
- SI3: Retained bacteria cooperation keywords
- SI4: GOs identified by annotating all MIDAS database genomes (5944 genomes) with PANNZER2.
- SI5: Full list of potential bacterial cooperation GO terms and description of manual curation decisions.
- SI6: Final list of bacterial cooperation GO used for the analysis
- SI7: Genomic diversity of the bacterial population within and across host. Computed from MIDAS snp_diversity.py pipeline.
- SI8: final dataset for statistical analysis.
- SI9: per-gene annotation of cooperation.
